# Nursing staff’s and physicians’ acquisition of competences and attitudes to interprofessional education and interprofessional collaboration in pediatrics

**DOI:** 10.1186/s12909-020-02128-y

**Published:** 2020-07-02

**Authors:** Christine Straub, Andrea Heinzmann, Marcus Krueger, Sebastian F. N. Bode

**Affiliations:** 1 Center for Pediatrics – Department of general pediatrics, adolescent medicine, and neonatology, Medical Center, Medical Faculty, University of Freiburg, Mathildenstrasse 1, 79106 Freiburg, Germany; 2 Department of Neonatology, Harlaching, Munich Municipal Hospitals, Munich, Germany

**Keywords:** Interprofessional collaboration, Interprofessional education, Interprofessional Questionnaire, Competence acquisition, Pediatrics

## Abstract

**Background:**

Interprofessional education (IPE) is deemed essential for interprofessional collaboration (IPC) in healthcare systems. IPC has positive effects for both patients and healthcare professionals. Especially in pediatrics, IPC is paramount for adequate care of patients and their families though there is a lack of data on the attitudes towards IPE and IPC and acquisition of respective competences in pediatric nursing and medical staff.

**Methods:**

Frequencies of interactions and attitudes towards IPE and IPC, with a focus on acquisition of competences for IPE and IPC, of nurses (*N* = 79) and physicians (*N* = 70) in a large pediatric university hospital were evaluated with an online questionnaire.

**Results:**

All participants worked as part of interprofessional teams, mostly consisting of nurses and physicians. The majority (94.9% (*n* = 75) of nurses and 100% (*n* = 70) of physicians) highly valued IPC. Medical doctors acquired most competences important for IPC during day-to-day work and reported a substantial lack of IPE. Nursing staff on the other hand did report significant interprofessional education during their training as well as ongoing interprofessional learning during day-to-day work. Nurses also appreciated IPE more.

**Conclusions:**

Even though IPC is commonly reported in nurses and physicians working at a large pediatric university hospital there is a lack of structured IPE. A focus should be on IPE for nurses and physicians to enable them to effectively collaborate together. Political and local initiatives for IPE are gaining momentum but still need to be established nationally and internationally.

## Background

Interprofessional collaboration (IPC) “… takes place when health professionals with different professional backgrounds work with patients, families, and caregivers to ensure the highest quality of care…” [1]. IPC is a mainstay of today’s healthcare [2]. It has been shown that efficient IPC can improve patient outcomes, reduce healthcare costs, and improve healthcare providers’ work satisfaction [3–6]. Especially in pediatrics the interaction of healthcare professionals with patients and their families as well as the collaboration of different healthcare professionals, are crucial for patient care [7–10].

Interprofessional education (IPE) has been defined as students from two or more professions learning together, about, and from each other [1]. IPE can lay a foundation for later effective IPC, especially regarding the procurement of essential skills and competences needed for interprofessional work [1, 11–16].

For effective IPC individuals need to acquire a multitude of different competences. Different frameworks have identified competences including values and ethics in IPC, team-based practice, communication skills, and others as essential for IPC [17–20]. The CanMeds physician competency framework for the first time strengthened the role of physicians as members of an interprofessional healthcare team [18]. Since then this concept has been adopted to different national frameworks for different health care professions [17–23]. Healthcare education still takes place monoprofessionally though in many countries, including Germany [24], but IPE is being integrated in more and more undergraduate and postgraduate curricula worldwide [25–27]. Therefore future health care professionals might be better prepared for their later work in interprofessional teams.

A lack of IPE and IPC has been demonstrated for both physicians and other health care professionals before [28–30]. In a previous study that focused on health care professionals other than nurses our group could show that physicians mostly received training regarding IPC while working [10]. As in a hospital setting the most common interprofessional interaction is that between medical and nursing staff [31], we decided to expand the study and to invite nurses of the same pediatric university hospital to participate.

The study was designed to evaluate pediatric nurses’ and physicians’ frequency of interactions with other health care professionals, attitudes towards IPE and IPC and to investigate the self-reported acquisition of competences required for IPE and IPC.

## Methods

### Questionnaire construction

We reviewed existing questionnaires which have been developed for measuring attitudes towards IPC including the Readiness for Interprofessional Learning Scale (RIPLS) [21], the Generic Role Perception Questionnaire (GRPQ) [22], the Interdisciplinary Education Perception Scale (IEPS) [23], and the Index of Interdisciplinary Collaboration (IIC) [24, 25]. Not all of the questionnaires were validated in German and none covered essential aspects that we deemed important: Most instruments lacked detailed questions regarding specific competences for IPE/IPC. Competences laid out in different IPE/IPC frameworks were reviewed [17, 18, 22] and added to our own questionnaire. Previous questionnaires did not differentiate if certain competences were acquired during education or while working. Additionally we put an emphasis on a short questionnaire. Therefore, we developed a new questionnaire focusing on the acquisition of competences, for both undergraduate studies and work. Nineteen items were selected in order to construct the questionnaire. Answers were graded from “1 = very frequently to 5 = never” for three questions regarding frequency of IP interactions and “1 = totally agree/very important/very helpful to 5 = strongly disagree/very insignificant/very hindering” for ten questions. Two questions regarding opportunities for IP learning were graded from “1 = too many to 6 = none”. Two open ended questions were included: “What are your wishes for the future of interprofessional collaboration?” and “What are your wishes for the future of interprofessional education?” The remainder of the questions comprised boxes to tick. Six items on demographics of the study participants were also included. Detailed data on the questionnaire construction and the questionnaire itself have been published before [10]. The questionnaire, including a list of competences assessed in this study, is available here: https://www.egms.de/tools/download.jsp?path=journals/zma/2016-33/zma001016.a1en.pdf&mime=application/pdf&name=Attachment_13.08.15.pdf.

### Study design

A cross-sectional study using convenience sampling was conducted. The questionnaire was provided electronically (Questback GmbH. Published 2015. EFS survey, version 10.5., Cologne, Germany) to be filled in online. All nurses and physicians at a large pediatric university hospital in Germany (Center for Pediatrics Freiburg – ZKJ) were invited to participate. Participants were able to fill in the questionnaire over a period of four weeks (physicians July 1st, 2014 to July 31st, 2014; nurses November 4th, 2015 to December 5th, 2015). A reminder email was sent after two weeks of initiation of the study.

### Study participants

A total of 79 nurses (response rate 37.3%) and 70 physicians (response rate 58.4%) participated in the study. The physicians were part of a larger cohort reported before [10]. Table 1 shows the demographics of the study participants.

**Table 1:** Demographics of the study participants. N/A = not applicable, no = number

Participants	Nurses	Physicians
	no (%)	no (%)
Invited to participate	212	120
Completed the questionnaire	79 (37.3)	70 (58.4)
Gender		
Female	72 (91.1)	39 (55.7)
Male	7 (8.9)	31 (44.3)
Work experience		
≤ 4 years	30 (37.9)	19 (27.1)
4–10 years	25 (31.6%)	26 (37.1)
≥ 10 years	37 (46.8)	25 (35.7)

### Data protection and ethical considerations

The study was conducted according to national data protection regulations. All data were collected anonymously. The employees’ committee of the University of Freiburg, Germany, approved the study. In addition, the ethics committee of the University of Freiburg, Germany, waived the need for ethical approval for this study.

### Data analysis

Descriptive statistics (e.g., mean and standard deviation) were used to describe the demographics of the study participants and survey data. Inferential statistics (e.g., unpaired t-tests, Kruskal-Wallis-tests) were used to determine differences and Bonferroni correction for multiple testing was applied as appropriate. Survey data were analyzed using SPSS version 23.0. Qualitative evaluation of the open-ended responses was performed according to the content analysis after Mayring [32]. One open-ended question focused on the future of IPC, one question was used to assess future needs for IPE. Participants’ answers to the open ended questions were analyzed using a deductive approach. Categories identified were structure/time, social skills/competences, interaction of different health care professionals, and conflict management/error culture for both IPC and IPE. The Figure was generated using GraphPad Prism version 7.01, GraphPad Software, La Jolla California USA, www.graphpad.com.

## Results

### Interprofessional collaboration

Overall, 94.9% (*N* = 75) of nurses and 100% (*N* = 70) of physicians either “highly valued” or “very highly valued” IPC. Physicians reported to work more frequently with psychologists, social workers and therapeutical educationists (Table 2). There were no significant differences between nurses and physicians in working together with physiotherapists or school teachers.

**Table 2:** Significant differences in frequencies of interactions of nurses and physicians with other health care professionals. Answers graded vom “1 = very frequently to 5 = never”. *Therapeutical educationists support children with special needs and their families

Item: How often do you work together with…		Psychologists	Nursery school teachers	Social workers	Therapeutical educationists*
Nurses		*M* = 2.58, *SD ±* .856	*M* = 3.73, SD ± 1.13	*M* = 2.94, *SD ±* .88	*M* = 3.96, *SD ±* 1.09
		t_(147)_ = 5.21, *p* < .001	*t*_(147)_ = 2,16, *p* = .032	t_(147)_ = 3.82, *p* < .001	*t*_(147)_ = 3.71, *p* < .001
Physicians		*M* = 1.93, *SD ±* .64	3.37, *SD ±* .89	*M* = 2.4, *SD ±* .82	*M* = 3.30, *SD ±* 1.08
Item: How often do you make decisions in the IP team together with…	Physicians	Psychologists	Teachers	Social workers	Therapeutical educationists
Nurses	*M* = 1.51, *SD ±* .69	*M* = 3.19, *SD ±* 1.05	*M* = 4.25, *SD ±* .93	*M* = 3.89, *SD ±* .96	*M* = 4.23, *SD ±* .99
	*t*_(147)_ = 4.92, *p* < .001	*t*_(147)_ = 6.65, *p* < .001	*t*_(147)_ = 2.49, *p* = .014	*t*_(147)_ = 5.84, *p* < .001	*t*_(147)_ = 3.24, *p* = .001
Physicians	*M* = 1.07, *SD ±* .26	*M* = 2.16, *SD ±* .81	*M* = 3.89, *SD ±* .86	*M* = 2.83, *SD ±* .93	*M* = 3.69, *SD ±* 1.06

Concerning patient care, physicians reported more frequently to reach joint decisions with other physicians, psychologists, social workers, and therapeutical educationists than did nurses (Table 2). There were no significant differences between nurses and physicians in in the frequency of involvement in the decision making process with other nurses, physiotherapists and nursery school teachers. Both professional groups most frequently interacted with nurses and physicians.

The collaboration with other physicians for treatment success was deemed more important by physicians (Mean (*M)* = 1.01, standard deviation (*SD) ±* .12) than by nurses (*M* = 1.24, *SD ±* .65*, t*_(147)_ = 2.89, *p* = .032). There were no differences in the appreciation for other health care professions regarding treatment success.

Physicians (*M* = 1.09, *SD ±* .28) rated IPC more important than nurses (*M* = 1.24, *SD ±* .54*), t*_(145)_ = 2.16, *p* = .032). There were no significant differences regarding additional qualifications, having a board of pediatrics certification, and participant-rated importance of IPC regarding the reported importance of IPC.

### IPE and acquisition of competences

**Fig. 1:**
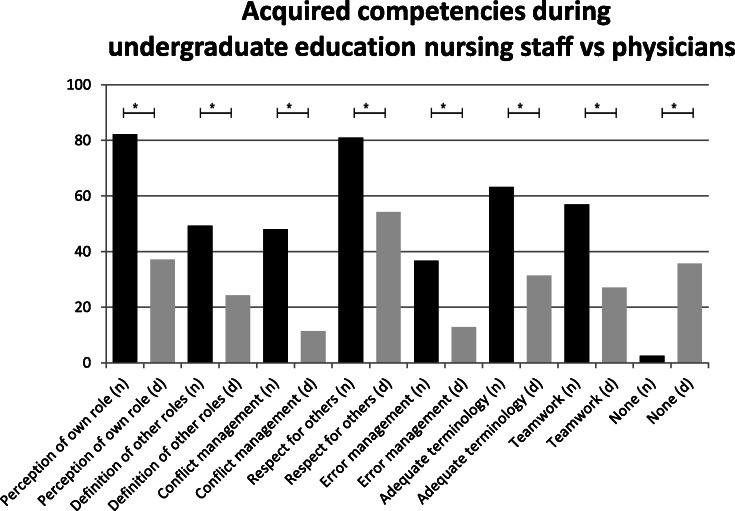
Acquisition of competencies during work and undergraduate education by nurses and doctors. Different competencies are displayed under the figure. Bars indicate the percentage of participants who stated to have acquired a competency. (d) = doctors; (n) = nurses. *: *p* < .05

Nurses reported significantly more opportunities for IPE during undergraduate training [(*M* = 3.89; *SD ±* 1.22) rather than during work (*M* = 4.28; *SD ±* 1.12); *t*_(78)_ = 2.16, *p* < .001]. However, physicians experienced less IPE during medical school (*M* = 4.73; *SD ±* 1.03) than during work (*M* = 3.94; *SD ±* 1.19, *t*_(69)_ = 4.17, *p* < .001). Concerning IPE, physicians had significantly fewer opportunities to learn together with other health care professionals than nurses during undergraduate studies (t_(147)_ = 4.52, *p* < .001). Physicians reported to have acquired more interprofessional competences through work experience rather than through undergraduate studies. In contrast, nurses acquired interprofessional competences while they were studying (Fig. 1). Both professional groups reported equal opportunities for IPC and interprofessional competence acquisition while working (t_(147)_ = 1.77, *p* = .078). Nurses (*M* = 1.81; *SD ±* .68) in general valued IPE higher than physicians (*M* = 2.27; *SD ±* .98, *t*_(149)_ = 3.37, *p* < .001).

### Routine interprofessional collaboration

Nurses reported both handlings of role conflicts and the appreciation of other professional groups more critically than did physicians. Nursing staff also stated that all relevant professional groups should be involved in the decision making process. Physicians thought more frequently that a doctor should lead the interprofessional team (Table 3). Work experience did not have an influence on perceived IPC.

**Table 3:** Significant differences in attitudes toward IPC depending on profession. Answers graded “1 = totally agree to 5 = strongly disagree”

	Nurses	Physicians	
	Mean (SD)	Mean (SD)	t/p-value
All relevant professional groups should be involved in the decision-making process.	1.33 (*±* .59)	1.71 (*±* .8)	*t*_(143)_ = 3.36, *p* = .001
Role conflicts in interprofessional teams are handled adequately in everyday work.	3.51 (*±* 1.06)	3.06 (*±* .87)	*t*_(143)_ = 2,81, *p* = .006
The work of other professional groups is not appreciated enough during every day work.	1.87 (*±* 1.04)	2.27 (*±* 1.02)	*t*_(143)_ = 2.35, *p* = .02
An interprofessional team should be led by a medical doctor.	3.63 (*±* 1.06)	2.34 (*±* 1.02)	*t*_(143)_ = 7.53, *p* < .001

### Requirements for IPC

Nurses rated IPE (*M* = 1.77, *SD ±* .68) and communication skills (*M* = 1.04, *SD ±* .19) as more essential for IPC than did physicians (*M* = 2.23, *SD ±* .78, *t*_(142)_ = 3.37, *p* < .001 and *M* = 1.19, *t*_(143)_ = 2.97, *SD ±* .39, *p* = .003, respectively).

Work experience had no influence on the perceived importance of different requirements for IPC.

### Qualitative evaluation

Through content analysis based on Mayring [32], we identified areas that participants deemed most important for future efficient interprofessional collaboration and interprofessional education.

#### Qualitative evaluation - Interprofessional collaboration

Structure and time: Establishing structures that support interprofessional collaboration on the hospital wards were mentioned frequently. These circumstances include both time and structural changes during day-to-day-work, mentioned as “to call for interprofessional collaboration” and “to intensify IPC”, for example.

Social skills, interaction and competences: Participants aspired for certain social skills and attitudes, which are needed in everyday interaction. This includes tolerance, “to reduce prejudices”, and patience, “to be able to make decisions at the same pace”. Furthermore many participants emphasized the importance “to respect…” “other professional groups”, “for different educational levels and experiences” as well “in our dealing with each other and caring for the opinions of others”.

Conflict management and error culture: Participants stated to “not settle conflicts at a personal level” but “to address problems and misunderstandings”. A communication and error culture needs to be established as “information about patients is often not disclosed” and “criticism is taken personally very quickly”.

#### Qualitative evaluation - Interprofessional education

According to the participants’ statements, we identified main categories concerning the wishes for the future of the interprofessional education.

Structure and Time: Participants called for “more structure for interprofessional education with other disciplines” and “more and better integration” of IPE in the curriculum. Providing more time resources for IPE during undergraduate studies of physicians and training of nurses were essential for the participants.

Social skills, interaction and competences: Participants put a special focus on interaction including flat hierarchies. For the future of IPE the participants hoped for an efficient interprofessional collaboration both on the ward and during education. This included “more and early contact of different disciplines”, as well as “interprofessional cooperation and communication during training”.

## Discussion

This study was designed to assess pediatric nurses’ and physicians’ frequency of interactions with other health care professionals, attitudes towards IPE and IPC and to investigate the self-reported acquisition of competences required for IPE and IPC.

The majority of physicians and nurses, who participated in the study, valued the importance of IPC. All reported to work interprofessionally routinely – not surprising as IPC is an integral part of daily practice in healthcare systems [2, 8]. The most frequent interprofessional interaction reported was between nurses and physicians as has been reported before [31] – hinting that those health professions might be the most important ones when limited resources for IPE and IPC have to be distributed.

In our cohort physicians stated to work significantly more with other health care professionals and to include other health care professionals more frequently in the decision making process regarding patients than did nurses. The cultural setting of the study has to be taken into account here: In Germany, as in some other countries [33], physicians are responsible for the overall care of the patients and therefore have to gather information from all involved health care professionals. In other healthcare systems nursing staff has a much more central role in patient care than in Germany [34, 35]. Additionally different education systems, especially in nursing, exist [35]. Therefore our results cannot be generalized.

In our study nurses rated the value of IPE higher than physicians but no differences were observed regarding the value of IPC. Previous work show an unclear picture: Some studies showed that nursing students were more ready for IPE than medical students [36, 37]. Another study reported nurses perceived IPE and IPC less important than physicians [38]. Interestingly these differences vanished when learning together. IPE leads to appreciation of interprofessional learning by those involved [16, 39]. Additionally there is even evidence that different professional groups, students, and working professionals all value IPE and IPC [15]. This inconsistency in the literature may be due to the different populations and cultural settings but generally hints at an enthusiasm for IPE and IPC that is further supported by our data. Especially the qualitative data reported here show that participants explicitly wish for IPE and IPC.

The most important findings of our study are the differences in the perception of acquisition of competences among physicians and nurses. While physicians reported a significant lack of competence acquisition during undergraduate studies, nurses reported to have gained those competences during education. This hints at more pronounced implicit practice-based competence acquisition and a lack of explicit IPE for the acquisition of competences in medical students which is also supported by a review elsewhere [40]. Another explanation for this might be that there still is a lack of structured IPE for medical students and a more practice based education for nurses in Germany: Nurses experience a 3-year non-university training that involves early contact with patients and other healthcare professions. Training in medical schools in Germany lasts at least 6 years and students only become members of an interprofessional team in their final year, thus giving medical students few opportunities for IPE in the early stages of their studies.

The German NKLM, the Master Plan for Medical Education 2020, and the new draft for medical licensure regulation, as well as similar concepts e.g. in Canada, Switzerland, or the United Kingdom aim at integrating IPE earlier in undergraduate medical education [17–19, 21, 22, 41]. Similar concepts are being implemented in education regulations for other health care professionals [23].

Even though there have been considerable efforts to either implement reformed medical curricula and/or to designate more time and energy towards educating medical students in communication in Germany [42], our data suggest that physicians retrospectively assess their acquisition of skills and competences essential for IPC worse than do nurses with a different kind of education. Successful local projects (IP degree for health care professionals, IP child protection seminars, IPE wards (for examples see: [12, 43–48]) have targeted IPE at different German medical schools and might serve as positive examples for others both nationally and internationally – something that the participants of our study clearly wish for.

Of positive note physicians seem to catch up in the acquisition of competences for IPC. The self-perceived competence level of practicing nurses and physicians does not differ. This might be a reflection of an ongoing learning process to work competently as part of an interprofessional team. It does put additional workload on junior physicians though, who already have to continue to gain knowledge in their selective fields and have to cope with their new role [9, 21, 29]. Earlier acquisition of interprofessional competences might enable a more efficient start for junior physicians on the wards and can probably improve patient care as well [49]. It may be too early to note positive changes of the re-structuring of medical education or first local projects in Germany. At least in our cohort we were not able to determine differences between physicians who just had finished medical school and those who had been working for over 10 years – but we are just at the beginning of reforming health care education.

### Strengths and limitations

Limitations of the study include the relatively small sample size and the setting as a cross-sectional study with convenience sampling at a single institution. Also recruitment periods were different for physicians and nurses. As no intervention took place we think the possible impact on the results is considerably small. Caution regarding the results has to be taken as the study is built on a self-reported retrospective assessment of interactions and own competences. Reliability and validity of the instrument have not been tested.

Despite the limitations we think the work presented here holds some interesting aspects: This study is the first that evaluates attitudes of nurses and physicians to IPE and IPC in a German pediatric university hospital. The data underline that IPC is already part of day to day work in pediatrics but hint that more IPE could support an easier start for junior doctors in this communicative field of medicine. It would be worthwhile to follow-up medical students and nursing trainees prospectively to gain a more profound understanding of their acquisition of competences. Ideally competences should be measured after proposed standards [50–52] – which might be part of a prospective follow-up study.

## Conclusions

Both pediatric nurses and physicians value the importance of IPC in routine clinical practice in pediatrics. Physicians acquire more interprofessional competences through work experience rather than through undergraduate studies. In contrast, nurses acquire interprofessional competences while they are studying. Taken together these results suggest that more focus should be put on IPE in German medical schools to help junior physicians with the acquisition of competences important for IPC before they start working as members of interprofessional teams. Fortunately IPE seems to gain momentum both politically but also locally in Germany. However, further work needs to be performed to establish a prospective assessment of interprofessional competences of nursing and medical students who then progress to working professionals.

## Data Availability

The datasets used and/or analyzed during the current study are available from the corresponding author on request.

## Abbreviations

GRPQ: Generic Role Perception Questionnaire
IEPS: Interdisciplinary Education Perception Scale
IIC: Index of Interdisciplinary Collaboration
IP: Interprofessional
IPC: Interprofessional Collaboration
IPE: Interprofessional Education
M: Mean
N / n: Number of participants / answers
RIPLS: Readiness for Interprofessional Learning Scale
SD: Standard deviation
ZKJ: Center for Pediatrics Freiburg, Germany
